# A Secure and Efficient ECC-Based Scheme for Edge Computing and Internet of Things

**DOI:** 10.3390/s20216158

**Published:** 2020-10-29

**Authors:** Hisham AlMajed, Ahmad AlMogren

**Affiliations:** Department of Computer Science, College of Computer and Information Sciences, King Saud University, Riyadh 11633, Saudi Arabia; 438105079@student.ksu.edu.sa

**Keywords:** authenticated encryption, asymmetric cryptography, chosen cipher text attack, chosen plain text attack, edge computing, elliptic curve cryptography, encryption, internet of things, industrial internet of things

## Abstract

Recent growth in the Internet of Things (IoT) has raised security concerns over the confidentiality of data exchanged between IoT devices and the edge. Many IoT systems adopt asymmetric cryptography to secure their data and communications. A drawback of asymmetric cryptography is the sizeable computation and space requirements. However, elliptic curve cryptography (ECC) is widely used in constrained environments for asymmetric cryptography due its superiority in generating a powerful encryption mechanism with small key sizes. ECC increases device performance and lowers power consumption, meaning it is suitable for diverse applications ranging from the IoT to wireless sensor network (WSN) devices. To ensure the confidentiality and security of data and communications, it is necessary to implement ECC robustly. A special area of focus in this regard is the mapping phase. This study’s objective was to propose a tested and trusted scheme that offers authenticated encryption (AE) via enhancing the mapping phase of a plain text to an elliptic curve to resist several encryption attacks such as Chosen Plaintext Attack (CPA) and Chosen Ciphertext Attack (CCA). The proposed scheme also undertakes evaluation and analysis related to security requirements for specific encryption attributes. Finally, results from a comparison of the proposed scheme and other schemes are presented, evaluating each one’s security characteristics and performance measurements. Our scheme is efficient in a way that makes so suitable to the IoT, and in particular to the Industrial IoT and the new Urbanization where the demands for services are huge.

## 1. Introduction

**Figure 1:**
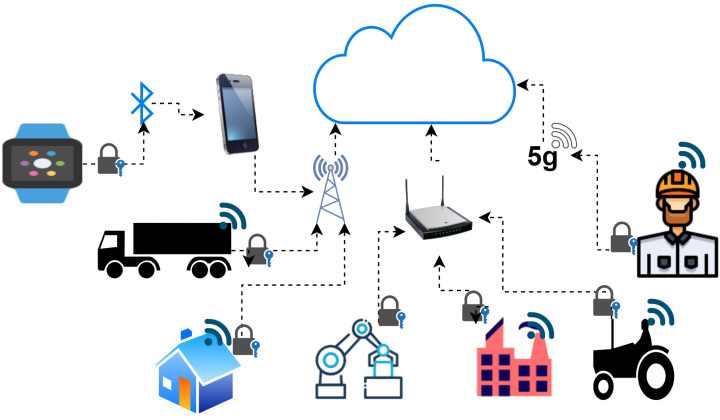
New technologies raised by the growth of industrialization and urbanization.

The continuous growth of industrialization and urbanization in recent years has led to the estimate that by 2025 there will be 21.5 billion actively connected Internet of Things (IoT) devices worldwide [1] as depicted in Figure 1. This remarkable growth of IoT shows that edge computing is increasingly used in today’s society [2]. In particular, the constrained properties of IoT devices, which include low performance in terms of computational resources and storage space, led to the adoption of edge computing [3]. For these reasons, the need to maintain data confidentiality and integrity has increased, which has caused an explosion of interest in cryptography schemes [4]. There are two types of encryption scheme, which are based on the nature of the key that will be used to encrypt and decrypt the data: which known as, symmetric cryptography and asymmetric cryptography [5]. The first type uses a one key for encryption and decryption, and it is useful if the two parties know the key before exchanging data. However, if the sender and the recipient cannot agree on a secure secret key exchange, then asymmetric cryptography is needed. In asymmetric cryptography, elliptic curve cryptography (ECC) is known for its superiority in producing a powerful encryption mechanism with small key sizes [6]. Therefore, ECC plays a valuable role in securing communications in constrained and resource-limited environments, including the IoT and wireless sensor networks (WSNs) [7].

### 1.1. Encryption Attacks on Asymmetric Cryptography

Information security consists of three aspects: confidentiality, integrity, and availability (CIA) [8,9,10]. Confidentiality and integrity are assured via cryptography schemes. One such scheme is asymmetric cryptography, which uses two key types: a public key and a private key [11]. The public key is used by the sender to encrypt the plain text, which gives it its name. This key is publicly available for use by anyone. By contrast, the recipient uses the private key to decrypt the received cipher text, which means that it must be known only by the recipient.

Asymmetric cryptography settles the dilemma of securing a shared key between two parties. At the same time, however, asymmetric cryptography suffers from a drawback in the key sizes used to encrypt and decrypt messages. Larger key sizes correspond to larger computation overheads for encrypting and decrypting plain text. However, ECC provides the same level of security as the Rivest–Shamir–Adleman (RSA) algorithm with short keys [12]. Therefore, ECC has emerged as the preferred approach for solving key size issues and for maintaining performance in constrained environments [13].

Many known attacks weaken existing cryptography schemes. They exploit vulnerabilities in the encryption process. For instance, the Known Plain Text Attack (KPA), Chosen Plain Text Attack (CPA), Cipher Text Only Attack (COA), and several types of Chosen Cipher Text Attack (CCA) have been identified. The KPA occurs when an attacker obtains a plain text and its corresponding cipher text. Specifically, the attacker attempts to obtain the encryption key [14]. The CPA occurs when an attacker selects random plain texts and requests the corresponding cipher texts for each text. Thus, the attacker aims to reduce the security of the scheme by analyzing both the plain text and cipher text [15]. In COA, the assumption is made that an attacker only has access to a set of cipher texts, where they can extract the secret key and/or the plain text [16]. The final attack, the CCA, involves the attacker gaining information by obtaining a sample of decrypted cipher texts of his/her choice [17].

In this research, the following attacks are used for the security analysis phase, denoted as [18]:

- IND-CPA: Indistinguishable under chosen plain text attack
- IND-CCA: Indistinguishable under chosen cipher text attack

### 1.2. Elliptic Curve Cryptography

The first curve used in elliptic curve cryptography (ECC) was introduced by Koblitz in 1987 [19]. ECC is widely used for devices in constrained environments, including the IoT and WSN devices. This stems from ECC’s value in affording the same level of hardness in terms of encryption as other asymmetric cryptography schemes, with significantly smaller key sizes and a lower computation overhead. As an illustration, the complexity of encryption in the RSA algorithm with a 1024-bit key is the same as the ECC algorithm with a 160-bit key. This noticeable difference in key sizes reduces the requirement for computation, and it lowers the storage needed to perform encryption. As a result, ECC supports low computation device capabilities, enabling them to perform more effectively [20]. ECC applications vary in the ways keys are exchanged between senders and recipients, as well as the approach used to secure communications between the two parties. In addition, ECC maintains message integrity by signing plain texts to prevent forgery.

The elliptic curve group operations are denoted as “+” for the addition of two points. For instance, let $P=(x_{1},y_{1})$ and $Q=(x_{2},y_{2})$, Therefore, the addition of P+Q can be expressed as $\left(x_{1},y_{1}\right)+\left(x_{2},y_{2}\right)=\left(x_{3},y_{3}\right)$. In some cases, where P=Q, the group operation is denoted as multiplication α×P on elliptic curve over $Z_{p},\;p>3$ that satisfy Equation (1), such that $a,b\in Z_{p}$ and $4\times a^{3}+27\times b^{2}\neq 0\;mod\;p$ where α is an integer value. For instance, $P+P=\left(x_{1},y_{1}\right)+\left(x_{1},y_{1}\right)=2P)$, similarly, 3P is equal to $\left(x_{1},y_{1}\right)+\left(x_{1},y_{1}\right)+\left(x_{1},y_{1}\right)$, and so on. It is important to note the the EC must be nonsingular curves (i.e., have no multiples roots).

(1) $$y^{2}\equiv x^{3}+a\times x+b\;mod\;p$$

The main operation in elliptic curves is the group multiplication operation [21,22,23,24]. For dP, this refers to *d* times of the addition of point *P*, which results in a new point $\left(x_{j},y_{j}\right)$. The private key is a large integer *d*, and the value of the multiplication operation $\left(x_{j},y_{j}\right)$ is known as the public key. The hardness of ECC is caused by the hardness of the mathematical problem, which states that, by knowing the public key point $\left(x_{j},y_{j}\right)$ and the starting point *P*, it is not possible to compute *d* in polynomial time [25]. This hardness is known in the literature as the elliptic curve discrete logarithm problem (ECDLP).

Several phases are involved in ECC to secure communications and encrypt transmitted data [26,27,28]. These phases are used together and/or separately, and they are listed as follows:

- Initializing system parameters
- Converting text values to numerical values
- Mapping numerical values to the elliptic curve
- Encrypting mapped points
- Hashing the message (for signing)

Similarly, the decryption phases are listed below:

- Verifying integrity of received message (signature verification)
- Decrypting cipher text
- Decoding mapped points to numerical values
- Converting numerical values to text values to represent plain text

The main computation process involved in the first phase is the calculation of the public keys derived by the multiplication of *d* (i.e., the private key) and *G* (i.e., the base point) [29,30,31,32]. The following phase involves the conversion of the plain text into numerical values, which is required because ECC depends on the use of numbers [33]. Therefore, the text must be securely converted in order to resist encryption attacks. Similarly, the third phase involves mapping the encoded value $x_{i}$ to the generated elliptic curve to find corresponding value of $y_{i}$ such that $\left(x_{i},y_{i}\right)\in E_{p}\left(a,b\right)$. If the mapping process fails in the first round, then $x_{i}$ is incremented by 1 until mapping is successful [34,35,36,37]. In the encryption phase, the cipher text is combined with the mapped point and secret key point $\left(x_{mapped},y_{mapped}\right)+\left(x_{key},y_{key}\right)$. The fifth phase maintains the integrity of the cipher text and ensures the sender’s nonrepudiation [38,39,40]. This is achieved by signing the cipher text using the sender’s signature, and it depends on the following steps [41,42,43]:

- Compute *r*, s.t. *r* is the $x_{R}\;mod\;p$ of $(x_{R},y_{R})=k*G$, where *k* is a random number and *G* is a base point
- Compute e=HASH(ciphertext) and obtain z=leftmostpbitsofe
- Compute *s*, where $s\equiv \left(d+z*r\right)k^{-1}\;mod\;p$, and where *d* is the sender’s private key
- The sent message is (ciphertext,(r,s))

Having provided an introductory overview, the rest of this paper is organized as follows: a literature review of other schemes is provided in Section 2; in Section 3, the details of the proposed scheme are described; in Section 4, security analysis and performance evaluations are presented in detail; and finally, concluding remarks and future research are discussed in Section 5.

## 2. Related Works

Elliptic curve cryptography (ECC) is frequently used to reduce the computational overhead caused by the limited capabilities of devices in constrained environments. Many schemes use ECC to secure communications between two parties by safeguarding the shared key exchange process. In particular, the elliptic curve integrated encryption scheme (ECIES) first employed the asymmetric approach by generating the shared key between two parties using ECC, after which the plain text was encrypted using the symmetric approach under the AES scheme [44,45,46,47]. On the other hand, many of these schemes failed to provide detail on how ECC was used to secure the plain text and/or how they were encoded into numerical values for use in ECC’s mapping phase.

Various proposed systems have enhanced key elements of the encryption process in ECC, but gaps have been identified in the literature. For instance, schemes proposed in [48,49,50] employ ECC without providing sufficient detail about how the plain text was encoded and mapped onto an elliptic curve. Therefore, enhancing these schemes tended to focus on performance rather than security. Similarly, [51,52,53] provided efficient algorithms for scalar multiplication in ECC that speed up the multiplication process. However, many schemes focus on securing ECC, and they offer more insights into the approach used to encode plain text and map it onto an elliptic curve. The rest of this literature review focuses on these schemes and, in particular, on the question of how the plain texts are encoded into numerical values, in addition, how these values are mapped onto the elliptic curve.

Several schemes proposed ways to secure plain text by encoding the characters to numerical values, thus giving them the ability to be mapped onto the elliptic curve. Many approaches use these schemes to encode the plain text. For instance, using the ASCII table, each character is converted into its decimal number [26,54,55,56]. In this case, the plain text “Hello” would be encoded to become “72” “101” “108” “108” “111”. These values are then mapped directly onto the elliptic curve as cipher text. However, this approach falls under the chosen plain text attack (CPA). This is because the attacker has the power to decrypt the chosen cipher text (in this case, a commonly used scheme). For this reason, other schemes manipulate the ASCII table by multiplying it by a secure number that is agreed on by both parties [57].

Critically, the issue of sharing the secure number is similar to the challenge of agreeing on the sharing of a secure key between the two parties. Such a scheme could also fall under the CPA and CCA. Similarly, other schemes are based on different encoding approaches that rely on matrix-based methods to conceal the matching table [58]. Specifically, these schemes use secret mapping tables that are unknown to anyone except the recipient, and which are used to encode and decode the plain text in a secure manner. While this may be true, there are two weakness associated with this approach: first, the dilemma of how to secure the delivery of the matrix table to the recipient in a secure way, thereby preventing CPA; and second, it is known that if the matrix-table is assumed to be securely delivered, then the cipher text falls under the CCA. This is because the same encrypted characters are repeated for the same plain characters in each encryption process.

The third approach to overcome flaws in ECC involves Block Chaining operations. The first step is to divide the plain text into a set of fixed-size blocks. In turn, the first block of the plain text is XORed with an initial vector InV [59]. Following this, the result of the first XORed value is used for the second XOR operation with a second block, and the process is repeated for all plain text blocks. This is a good approach, but it is vulnerable to CPA and CCA when the plain text is divided into a set of blocks and all blocks are same (e.g., the plain text is a repeated character). As a result, the second XORing process results in the value of the InV as following $InV⊕B_{1}⊕B_{2}=InV$. This is because $B_{1}=B_{2}$. Therefore, the result of the XORing operation becomes $B_{1}^{\prime },InV,B_{1}^{\prime },InV,...$, where $B_{1}^{\prime }=B_{1}⊕InV$. Additionally, this scheme lacks AE property, which increases vulnerability to cipher text tampering attacks and nonrepudiation issues.

Barman et al. [60] proposed an encryption method to secure IoT communications informed by DNA-based ECC. For each set of characters in the plain text, a DNA genome sequence is mapped to it. There are many genome sequences publicly available, and so randomizing the selection of genome sequences is part of the encryption process for the plain text. Furthermore, the decryption process should use the same DNA genome sequences. Therefore, both parties, the sender and the recipient, must use the same sequences before encrypting and decrypting the plain text. For this reason, the DNA genome sequences should be securely used only by the sender and recipient. If an attacker discovers the sequence, the scheme will be vulnerable to encryption attacks such as CPA. Additionally, even if the sequence is delivered securely, the cipher text will be the same for each repeated plain text encryption process. Resultantly, the scheme is also vulnerable to other encryption attacks, including CCA.

Duarah et al. [61] introduced Securing IoT Using Machine Learning and ECC. In their scheme, the authors first classified the data set to enhance the transmitted data, where the accurate data were transmitted only to reduce computation efforts. If the data were clean, then they were moved into a second stage, namely encryption via ECC. However, if the data is malicious then it is discarded to save the encryption computation efforts. In the encryption phase, the authors wrote the key generation algorithms that use the ECC scalar multiplication operation δ=(P)∗(d) s.t. *P* is a point in the elliptic curve, and *d* is the private random integer. In their encryption algorithm, the authors defined how the shared key is constructed in order to encrypt the plain text by the addition operation between the plain text and the shared key. Although the scheme produced a new strategy for performance enhancement in the IoT environment by limiting data transmission only to clean data, the security analysis indicates that it is vulnerable to CPA when the same data are encrypted and sent using the same scheme.

Joglekar et al. [62] proposed Lightweight ECC for Data Integrity and User Authentication in Smart Transportation IoT System. In their scheme, the authors use One Time Password (OTP) to exchange the shared key securely to prevent the man in the middle attack (MITM). The 4-digit OTP is encrypted using ECC and transmitted to the recipient to complete the registration process. The shared key between the two parties is constructed as follows: $S=d_{A}Q_{B}=d_{B}Q_{A}$, where $d_{A},d_{b}$ are private keys for Sender *A* and Recipient *B*, respectively. Similarly, $Q_{B}=d_{B}G$ and $Q_{A}=d_{A}G$ are the public keys for Sender *A* and Recipient *B*. Using OTP to prevent the MITM is a good approach, but the authors neglected to state whether there is an assumption about the 4-digit OTP. It is relevant that the brute force attack needs $10^{4}=10,000$ possible choices to break the OTP, which can be completed in several minutes. Furthermore, the authors did not state how to encrypt the OTP or how to map it onto an elliptic curve.

Finally, Das & Giri [55] proposed two encoding algorithms, which generate sets of numerical values via the sum of weight *n* with base *b* IntegerDigits[n,b]−1. The first encoding algorithm is used when the value of the base *b* is a dynamic integer. In this case, the highest accepted value is 65,536 (i.e., the highest value of ASCII table). In addition, *n* is the size of the prime field, where the authors suggest the use of a 192-bit key. Resultantly, the set of groups that can be combined based on their method is IntegerDigits[192bit,65,536]−1=11. The base *b* can be reduced below 65,536, which increases the set of groups more than 11 based on *b*. However, the reduction of *b* should also contribute to a reduction in ASCII table mapping, and the authors did not provide a safe reduction mechanism. When *b* is not dynamic in the second algorithm, the suggested set of the combing group is equal to $\frac{Number\;of\;p\;digits}{IntegerDigits[n,b]-1}$. Based on the scheme described by the authors, it is $\frac{58-1}{11}=6$, and the number of groups based on both algorithms is small. Thus, the computation overhead will increase compared to other schemes. Notably, the encoding and mapping phases did not manipulate the plain text characters after using the ASCII table values, which heightened the scheme’s vulnerability to CPA.

All schemes in the literature are vulnerable to CPA and/or CCA, such that the encryption of the same plain texts always produce same cipher texts. Besides, these schemes use the appending method in the mapping phase which results in increasing the computation overhead. Although, some schemes that use the probability method to map points to EC failed to justify the chosen value of *k*. Moreover, many of the existing schemes fail to offer secure AE scheme, which means they are vulnerable to tampering attacks. An attacker can modify the transmitted cipher text without detection by the receiver. The cipher text offers confidentiality only; it does not offer integrity by itself. Therefore, to prevent tampering, to maintain the integrity of the cipher text, to ensure that the transmitted message is secured against encryption attacks, and to enhance the mapping phase performance, the proposed scheme ensures the AE property, resists CPA and CCA, and enhances the mapping phase performance.

## 3. The Proposed Scheme

The proposed scheme contains nine phases: initializing system parameters; converting the plain text message into numerical values (encoding); finding the mapping points on the elliptic curve; encrypting the mapped points; signing the aggregated mapped points as cipher text; and finally, undertaking the reverse of the previous phases by verifying and decrypting the received cipher text, decoding the mapped points, and converting the mapped points into plain text.

**Figure 2:**
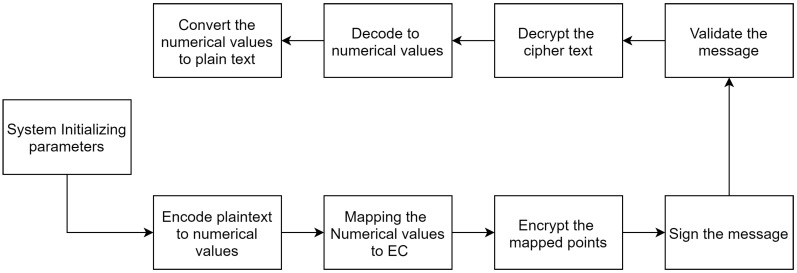
High-level overview of proposed scheme.

This study’s main contribution is to offer a secure and efficient encryption scheme in the form of ECC. It facilitates secure communication by creating a shared key for a group of parties, which they can use to secure their shared messages. Notably, shared key creation is undertaken in the first phase, and many recent studies neglect to highlight the importance of having a shared key between parties for shared message encryption. In addition, this study provides a secure and enhanced method to encode and map plaintext to EC. Therefore, this study focuses on the three phases that start the ECC because it constitutes a major feature of any system for securing group communication (e.g., IoT environments). Figure 2 shows the nine phases of the proposed scheme.

### 3.1. Generating System Parameters

The aim of this phase is to generate the parameters needed to secure communication between all parties by setting up public and private keys. This phase contributes to the creation of the shared group key $gk_{sh}$, which is used to encrypt the message shared between group members. The notations that the system generates, which are used for each session in the proposed scheme, are illustrated in Table 1.

It is important for the edge to create and maintain the shared group key $k_{sh}$. This enables the parties to decrypt cipher text effectively. Therefore, edge creates the initial $k_{sh}$ using its $H_{1}\left(id_{edge}\right)$ and PRK. The key generation process is illustrated in Algorithm 1.
**Algorithm 1:Edge algorithm for generating initial shared group key $k_{sh}$.**  **Input**: $id_{edge};\;H_{1};\;PRK$  **Output**: $k_{sh}$**1**  $k_{sh}=H_{1}\left(id_{edge}\right)⊕PRK$**;****2**  $Initial\;shared\;group\;key\leftarrow k_{sh}$

In a similar way, for every new node that joins the group, $k_{sh}$ is updated by the following Equation (2).

(2) $$k_{sh}=H_{1}\left(id_{ni}\right)⊕k_{sh}$$

To maintain the forward and backward secrecy, it is important to ensure that all new nodes that join cannot decrypt the cipher text sent before it joined the group using $k_{sh}$. Similarly, any nodes that leave the group cannot decrypt the cipher text sent after their departure. To achieve this, the proposed scheme allows each node to perform certain operations to maintain $k_{sh}$.

**Figure 3:**
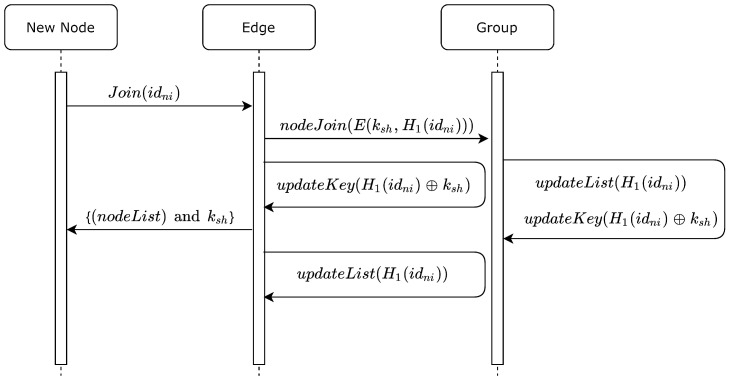
Sequence diagram for new node joining the group.

For a new node to join the group, the edge sends the joined node hashed ID $H_{1}\left(id_{ni}\right)$ to all current nodes. In turn, each node adds the hashed ID to its node list nList, and it simultaneously updates $k_{sh}$ using Equation (2). Similarly, the edge updates its $k_{sh}$ using the same equation. In turn, the edge sends $k_{sh}$ to the newly joined node, along with a list of all hashed IDs of existing node (with the exception of the hashed ID of the new node). Figure 3 illustrates this process, and Algorithm 2 describes the steps required to perform the joining process.
**Algorithm 2:Updating $k_{sh}$, $gk_{sh}$, and nList for a newly joined node.**  **Input**: $id_{ni};\;H_{1};\;k_{sh}$  **Output**: $Updated\;k_{sh}\;,gk_{sh}\;and\;nList$**1**  $New\;node\;requests\;join(id_{ni})$**;****2**  NewnodeandEdgestartMAprocess**;****3**  $Edge\;broadcast\;H_{1}\left(id_{ni}\right)\;encrypted\;by\;current\;gk_{sh}$**;****4**  $Edge\;and\;current\;nodes\;update\;key\;k_{sh}=H_{1}\left(id_{ni}\right)⊕k_{sh}$**;****5**  $Edge\;and\;current\;nodes\;update\;shared\;point\;gk_{sh}=k_{sh}\times G$**;****6**  $Edge\;sends\;k_{sh}\;and\;nList\;to\;new\;node$**;****7**  EdgeandcurrentnodesupdatetheirnList

**Figure 4:**
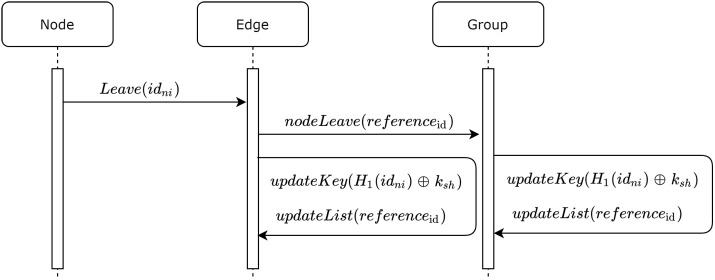
Sequence diagram for node leaving the group.

Similarly, when the current node leaves the group, the edge sends its reference ID to all currently joined nodes, and each node updates $k_{sh}$ using Equation (2). At the same time, the corresponding hashed IDs are removed from the node list. Additionally, the edge updates $k_{sh}$ using Equation (2), and it removes the corresponding hashed ID from its node list. Figure 4 presents a sequence diagram illustrating the process, and Algorithm 3 describes the steps required to perform the leaving process.
**Algorithm 3:Updating $k_{sh}$, $gk_{sh}$, and nList for node leaving the group.**  **Input**: $id_{ni};\;H_{1};\;k_{sh}$  **Output**: $Updated\;k_{sh}\;,gk_{sh}\;and\;nList$**1**  $Node\;sends\;leave(id_{ni})$**;****2**  $Edge\;broadcast\;nodeLeave(Enc\left(gk_{sh},reference_{id}\right))$**;****3**  $Edge\;and\;current\;nodes\;update\;key\;k_{sh}=H_{1}\left(id_{ni}\right)⊕gk_{sh}$**;****4**  $Edge\;and\;current\;nodes\;update\;shared\;point\;gk_{sh}=k_{sh}\times G$**;****5**  EdgeandcurrentnodesupdatetheirnList

### 3.2. Encoding and Mapping the Plain Text

**Figure 5:**
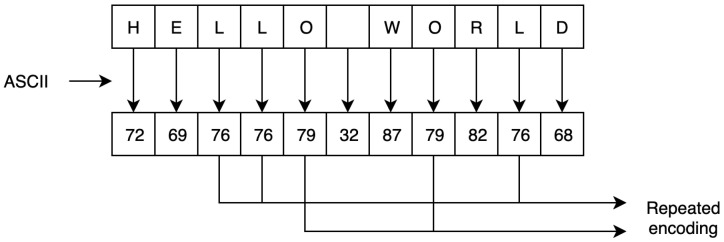
Repeated encoded characters using the ASCII table.

**Figure 6:**
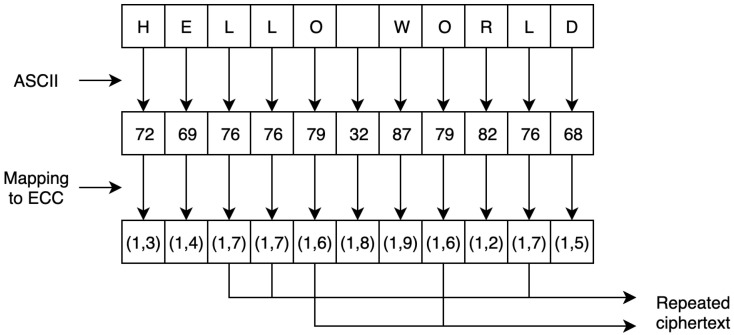
Repeated mapped points to the designated elliptic curve using the ASCII table.

This study extended the encoding and mapping mechanism steps proposed in [63]. As described in related work, a security flaw exists in the study’s encoding process. The main encryption flaw associated with the encoding and mapping phases in ECC stems from the fact that using the same encryption scheme produces the same ciphertext. As such, by analyzing the ciphertext, the adversary can gain important information about the plaintext. To illustrate this flaw, Figure 5 shows how the same letter is encoded with the same value every time. Thus, the corresponding ciphertext of the encoded value is transformed into the same ciphertext value each time, which allows the adversary to distinguish between the set of ciphertexts to extract the plaintext, as shown in Figure 6.

Every scheme discussed in this study’s literature review in Section 2 is vulnerable to an encryption flaw. Therefore, the study provides an improved encoding process to overcome this flaw by dividing the plain text into a set of blocks denoted by *B*. Then, the characters of each block *B* are counted, denoted by *N*, which can be calculated as follows:

(3) $$N\leq \;\lfloor \frac{p-8}{8}\rfloor$$

It is worth noting that 8 is subtracted from *p* to allow a maximum of 8 bits in terms of the padding bits used in the mapping phase. Therefore, in the proposed scheme, we can determine the value of *N* as follows:

(4) $$N\leq \;\lfloor \frac{192-8}{8}\rfloor =23$$

In a similar way, the number of blocks *B* needed in each plain text, denoted by the number of characters *M*, is determined by the following equation:

(5) $$B=\;\lceil \frac{M}{N}\rceil$$

**Figure 7:**
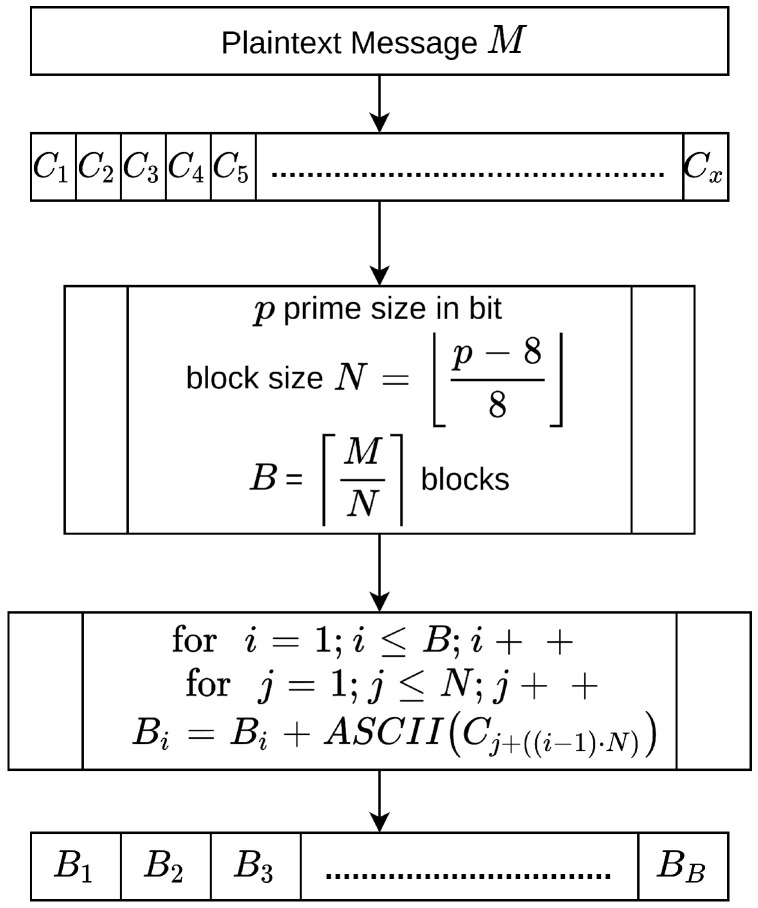
Converting plain text into a set of blocks.

The to divide the plaintext to this length is because every block *B* must be mapped to the 192-bit ECC (field size 192). As discussed previously, 8 bits in every block are reserved for the mapping phase, in which they serve as padding bits to secure the number of rounds required to find the mapping points for each block. Figure 7 illustrates the process needed to convert the plain text *M* into set of blocks.

Algorithm 4 provides an overview of the steps involved in converting plain text into a set of blocks.
**Algorithm 4:Converting a plain text into a set of blocks.**  **Input**: TheplaintextMandp  **Output**: Setofblocks**1**  Sender:obtaintheplaintextM**;****2**  Sender:calculatethesizeofeachblock**;****3**  $\;\;\;\;\;\;N\leftarrow \;\lfloor \frac{192-8}{8}\rfloor$**;****4**  Sender:calculatethenumberofblocks**;****5**  $\;\;\;\;\;\;B\leftarrow \;\lceil \frac{M}{N}\rceil$**;****6**  Sender:dividethepalintextintoBblocksofsizeN**;****7**  fori=1;i<=B;i++**;****8**  forj=1;j<=N;j++**;****9**  $\;\;\;\;\;\;\;Sender:\;obtain\;the\;B_{i}\;=\;B_{i}\;+\;ASCII\left(c_{j+((i-1)*N)}\right)$**;****10**  Setofblocks←theresults

**Figure 8:**
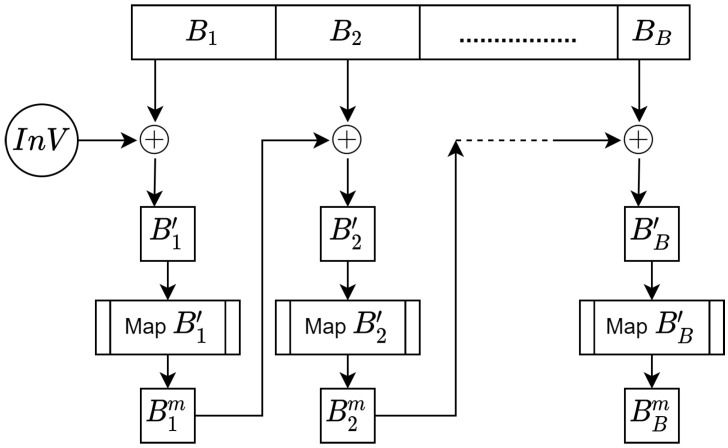
Securing blocks for resistance against encryption attacks using Cipher Block Chaining (CBC).

Following the conversion of *M* into a set of blocks, the next critical issue is to ensure that the blocks are secure against encryption attacks (e.g., CPA and CCA). Therefore, the Cipher Block Chaining (CBC) was used to make the blocks resistant to such attacks. Figure 8 shows the process of ⊕ the blocks to increase their security.

Algorithm 5 describes the process that secures the blocks.
**Algorithm 5:Securing blocks for resistance against encryption attacks.**  **Input**: $Blocks\;retrieved\;(B_{i})\;from\;message\;and\;InV$  **Output**: EncodedblockswithInV**1**  fori=0;i<noofblocksB;i++**;****2**  $\;\;\;\;let\;{B_{i}}^{\prime }=B_{i}⊕InV$**;****3**  $\;\;\;\;let\;{B_{i}}^{m}=map\left({B_{i}}^{\prime }\right)$**;****4**  $\;\;\;\;let\;InV={B_{i}}^{m}$**;****5**  $Encoded\;message\leftarrow the\;set\;of\;{B_{i}}^{\prime }$

**Figure 9:**
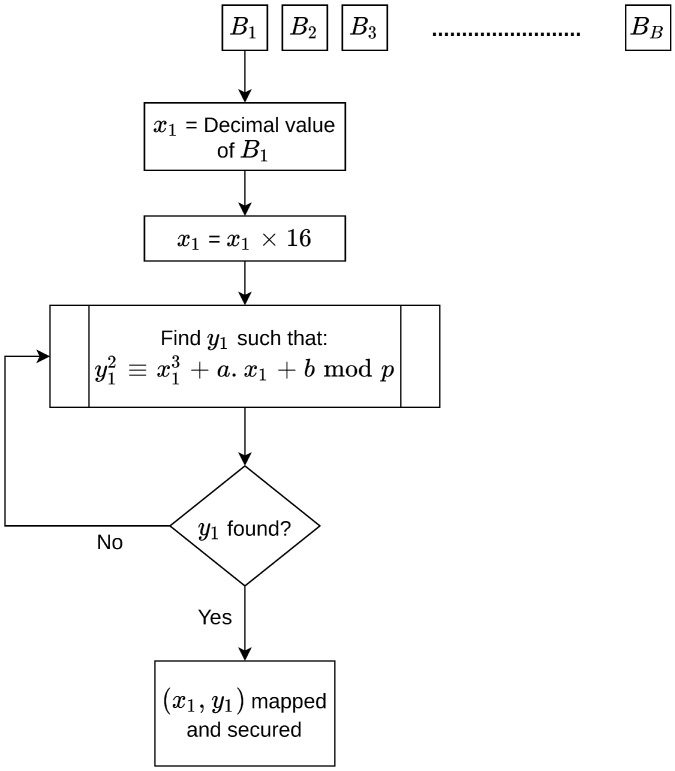
Mapping secured blocks to an elliptic curve.

Mapping a point to an elliptic curve means that $\left(x_{i},y_{i}\right)$ satisfies Equation (1). Therefore, it is important to find the value of $y_{i}$ corresponding to $x_{i}$ for each point. Every secured block is converted into a decimal value, and these values are then mapped to the elliptic curve generated in the first phase to find the corresponding value of $y_{i}$. Figure 9 illustrates the steps involved in mapping the secured blocks to an elliptic curve.

Algorithm 6 outlines the steps involved in mapping the secured blocks to an elliptic curve.
**Algorithm 6:Mapping secured blocks to an elliptic curve.**  **Input**: Securedblocks  **Output**: Mappedpoints**1**  fori=0;i<noofsecuredblocksB;i++**;****2**  $\;\;\;\;Sender:\;obtain\;the\;decimal\;value\;x_{i}\;for\;the\;secured\;block$**;****3**  $\;\;\;\;Sender:\;x_{i}\;=\;x_{i}\times 16\;$**;****4**  $\;\;\;\;Sender:\;obtain\;corresponding\;y_{i}\;using\;the\;equation$**;****5**  $\;\;\;\;Sender:\;if\;y_{i}\;cannot\;satisfy\;the\;equation$**;****6**  $\;\;\;\;Sender:\;x_{i}=x_{i}+1$**;****7**  $Sender:\;repeat\;the\;loop\;until\;find\;y_{i}$**;****8**  Mappedpoints←theresults

### 3.3. Encrypting and Decrypting the Mapped Points

**Figure 10:**
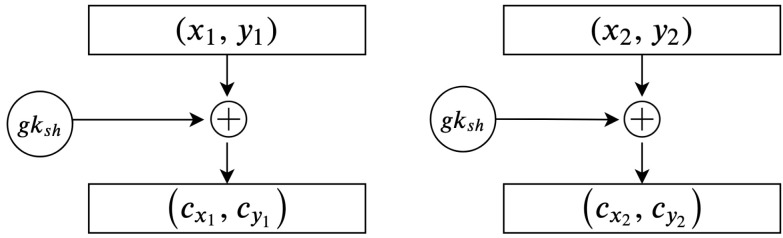
Encrypting mapped points using the shared group point.

In many schemes, the assumption is made that the mapping phase is sufficient to secure the plain text and plays the encryption phase. However, this assumption is incorrect because the mapping phase to the elliptic curve means that these points belong to the generated elliptic curve and can benefit from the elliptic curve discrete logarithm problem (ECDLP). In our proposed scheme, we encrypt these points by adding them to $gk_{sh}$. Figure 10 illustrates the process for encrypting the mapped points.

The algorithm used to secure the transmitted message using the shared group point $gk_{sh}$ is given below.
**Algorithm 7:Encrypting mapped points using the shared group point.**  **Input**: $Mapped\;points\;\left(x_{i},y_{i}\right),\;gk_{sh}$  **Output**: Ciphertexts**1**  $Sender:\;Calculate\;gk_{sh}$**;****2**  fori=0;i<noofmappedpointsB;i++**;****3**  $\;\;\;\;\;\left(c_{xi},c_{yi}\right)=gk_{sh}+\left(x_{i},y_{i}\right)$**;****4**  Sender:repeatuntilfinish**;****5**  $(c_{xi},c_{yi})\leftarrow the\;results$

**Figure 11:**
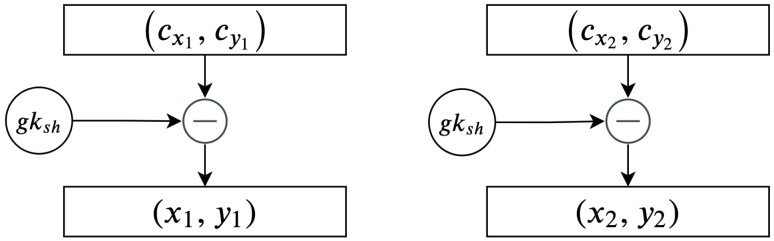
Decrypting secured points using the shared group key.

The remaining phases constitute a reversal of the previous phases. Therefore, the secured points are decrypted, and the recipient subtracts them with $gk_{sh}$. As illustrated before, all parties have the same shared group point $gk_{sh}$, which is used to encrypt and decrypt the messages. In Figure 11, an illustration of how to decrypt secured points is given.

Algorithm 8 provides an overview of the steps involved in completing the decryption process:
**Algorithm 8:Decrypting cipher texts using the shared group key.**  **Input**: $Encrypted\;points\;\left(c_{xi},c_{yi}\right),\;gk_{sh}$  **Output**: Decryptedpoints**1**  $Recipient:\;Calculate\;gk_{sh}$**;****2**  fori=0;i<noofEncryptedpointsB;i++**;****3**  $\;\;\;\;\;\left(x_{i},y_{i}\right)=\left(c_{xi},c_{yi}\right)-gk_{sh}$**;****4**  Recipient:repeatuntilfinish**;****5**  $(x_{i},y_{i})\leftarrow the\;results$

### 3.4. Decoding and Converting the Decrypted Points into Plain Text

**Figure 12:**
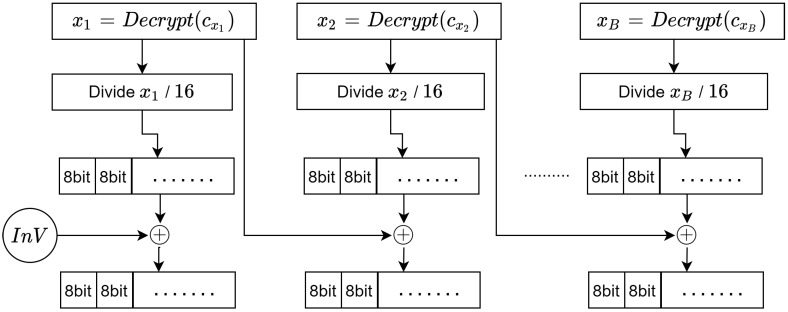
Converting and decoding $x_{i}$ values into binary values.

The next phases are concerned with completing the previous phase, which focus on decoding and converting the decrypted points into a plain text *M*. There are two pairs on each set of these points, denoted $x_{i}$ and $y_{i}$. It is worth noting that $y_{i}$ is used in two phases only: the encryption and decryption phases. In these phases, the values are used to add and subtract two points on the elliptic curve. Therefore, in this phase, we are only concerned with $x_{i}$, that represent the binary values of the characters in the plain text. The process that describes the decoding phase is illustrated in Figure 12.

Algorithm 9 demonstrates the steps required to convert the decrypted points $x_{i}$ into binary values:
**Algorithm 9:Decoding decrypted points into binary values.**  **Input**: $Decrypted\;points\;x_{i},\;InV$  **Output**: Binaryvalues**1**  $Recipient:\;obtain\;x_{i}\;value\;for\;the\;decrypted\;points$**;****2**  fori=0;i<noofdecryptedpointsB;i++**;****3**  $\;\;\;\;let\;InV_{save}=x_{i}$**;****4**  $\;\;\;\;let\;x_{i}=x_{i}÷16$**;****5**  $\;\;\;\;let\;x_{i}=x_{i}⊕InV$**;****6**  $\;\;\;\;let\;InV=InV_{save}$**;****7**  Recipient:repeatuntilfinish**;****8**  $Binary\;values\leftarrow the\;results\;x_{i}$

**Figure 13:**
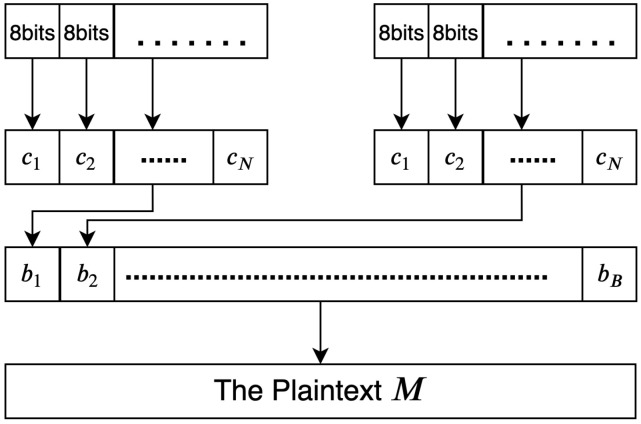
Converting binary values into plain text *M*.

Finally, the binary values obtained in the previous phase are converted into their corresponding characters. These binary values represent the plain text message *M*, and so they need to be converted into their ASCII values. The conversion process is illustrated in Figure 13.

Algorithm 10 shows how to convert binary values into a plaintext *M*.
**Algorithm 10:Converting binary values into plain text.**  **Input**: Binaryvalues,B  **Output**: TheplaintextmessageM**1**  Recipient:Getthebinaryvalues**;****2**  fori=0;i<noofbinaryvaluesB;i++**;****3**  converteach8bitsintoitscorrespondingASCIIcode**;****4**  foreachNcharaggregatetosingleblock**;****5**  Recipient:repeatuntilfinish**;****6**  ThemessageM←theresults

### 3.5. Signing and Verifying the Encrypted Message

**Figure 14:**
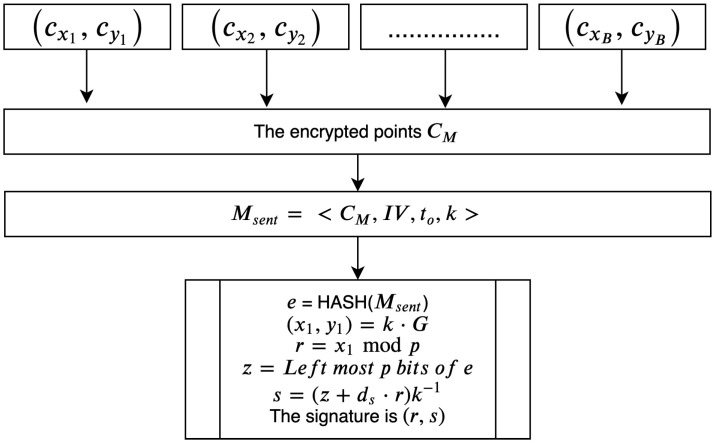
Sender ensures message integrity by signing it with $d_{s}$.

The intention of AE schemes using the encrypt then sign approach is to transmit messages between parties without compromising confidentiality, security, and integrity. In the proposed scheme, confidentiality (encryption) is maintained by the previous phases. Thus, to assure integrity (sign), the sender signs the message using his or her private key $d_{s}$, which relies on ECDSA. The message $M_{sent}$ consists of a set of tuples, which contain the encrypted points $C_{M}$ and InV, the timestamp $t_{o}$, and a random signature integer *k*. The signing process is illustrated in Figure 14.

The following algorithm outlines the process used to sign the $C_{M}$:
**Algorithm 11:Process of signing the message.**  **Input**: $The\;Message\;M_{sent}$  **Output**: Signaturepairs(r,s)**1**  $Sender:\;calculate\;e=HASH(M_{sent})$**;****2**  Sender:calculatez=leftmostpbitsofe**;****3**  Sender:selectrandomvaluek**;****4**  $Sender:\;calculate\;(x_{1},y_{1})=k\times G$**;****5**  $Sender:\;calculate\;r=x_{1}\;mod\;p\;where\;r\neq 0$**;****6**  Sender:forr=0goto3**;****7**  $Sender:\;calculate\;s\;=\;\left(z+d_{s}\;*\;r\right)\;k^{-1}\;for\;s=0\;go\;to\;3$**;****8**  Sender:(r,s)←ciphertextsignature

**Figure 15:**
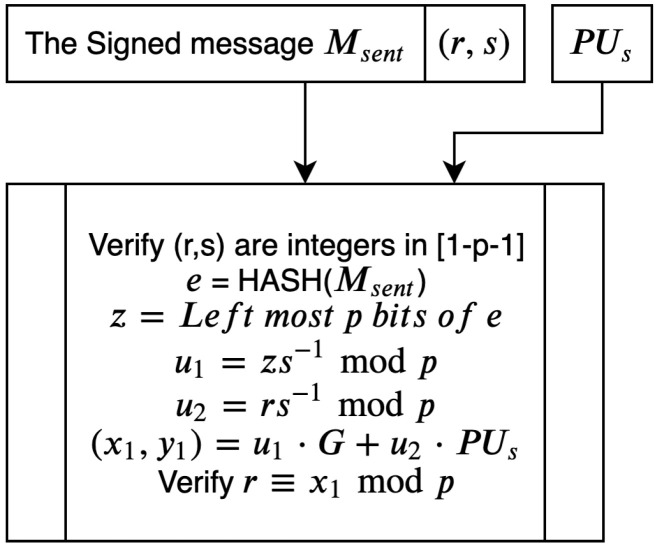
Receiver verifies signed message.

The receiver of a signed message verifies it using the sender’s public key. The verification process is illustrated in Figure 15.

Algorithm 12 outlines the steps involved in verifying the integrity of the received message using the sender’s public key.
**Algorithm 12:Recipient verification of a signed message.**  **Input**: $M_{sent},\;\left(r,s\right),\;PU_{s}$  **Output**: Verifiedciphertexts**1**  Recipient:check(r,s)areintegers∈1,2,3,...,p−1)**;****2**  $Recipient:\;calculate\;e=HASH(M_{sent})$**;****3**  Recipient:calculatez=leftmostpbitsofe**;****4**  $Recipient:\;calculate\;u_{1}=es^{-1}\;mod\;p$**;****5**  $Recipient:\;calculate\;u_{2}=rs^{-1}\;mod\;p$**;****6**  $Recipient:\;calculate\;\left(x_{1},y_{1}\right)=u_{1}*G+u_{2}*PU_{s}$**;****7**  $Verified\leftarrow \;r\equiv x_{1}\;mod\;p$

## 4. Security Analysis and Performance Evaluation

**Figure 16:**
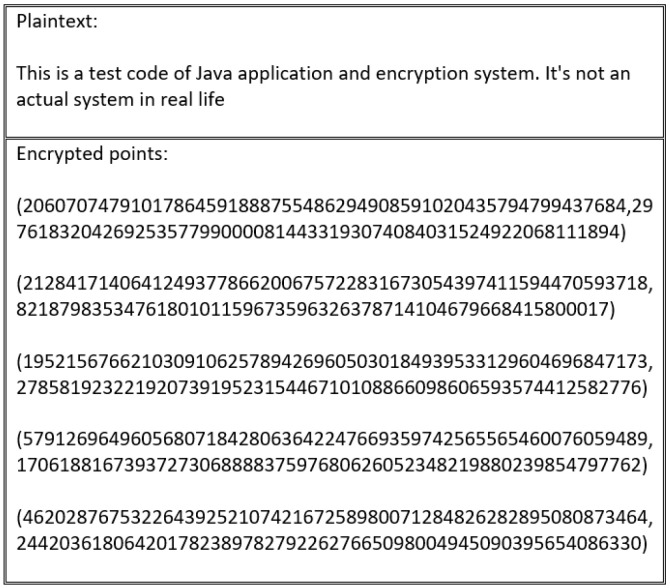
Cipher text generated by first encryption process.

**Figure 17:**
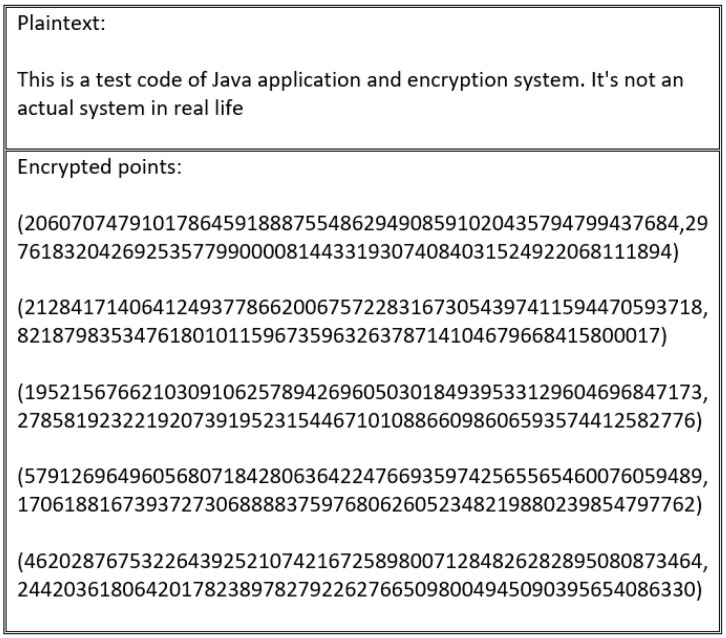
Cipher text generated by second encryption process using the same plain text as in Figure 16.

Every scheme discussed in this study’s literature review is vulnerable to an encryption flaw. For instance, the encryption schemes presented in [55,60,61,62] produce the same cipher text when repeated several times, as outlined in Figure 16 and Figure 17.

These schemes suffer from an encryption weakness because they offer the same cipher text if the sender encrypts the plain text using the same encryption key. The main factor that underlies this flaw is that the encryption process in these schemes neglects to consider the importance of manipulating plain text before encrypting and decrypting it. This ensures that a distinct cipher text is produced for every encryption process. Therefore, in the absence of a manipulation step, the adversary can learn from the cipher text, determining whether certain cipher texts are the same or different.

**Figure 18:**
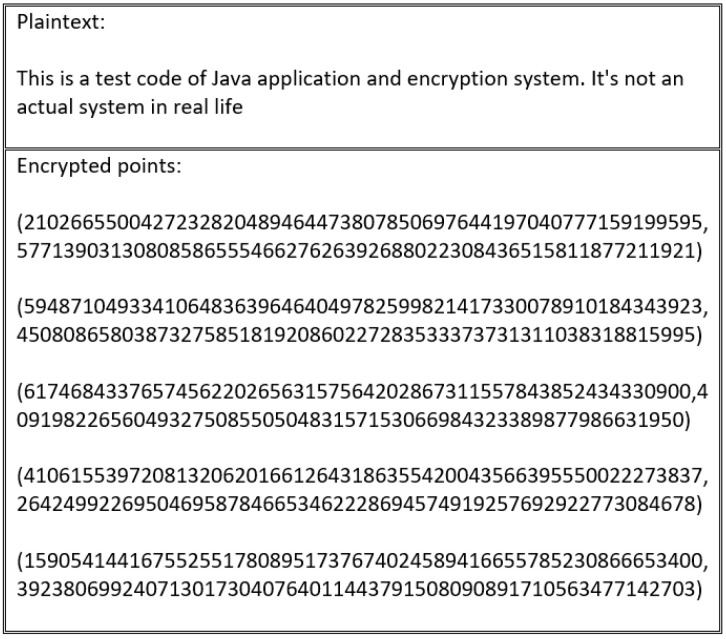
Cipher text generated by first encryption process using XOR with InV.

**Figure 19:**
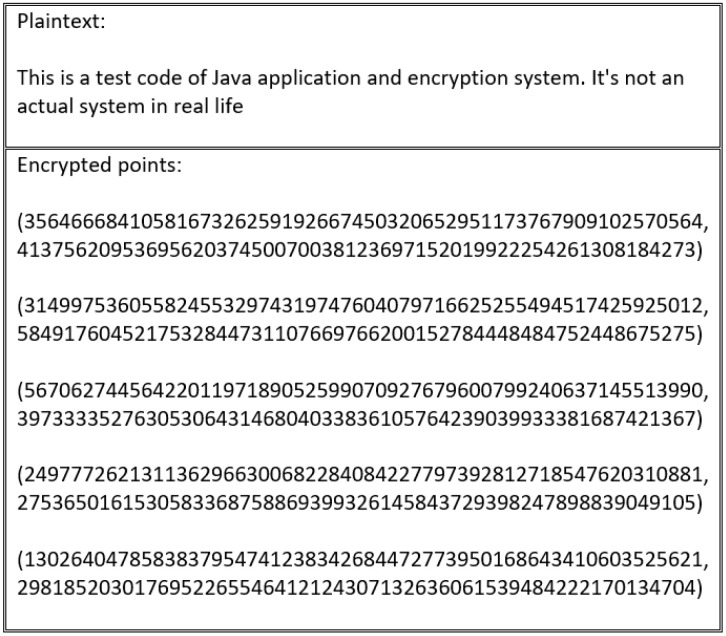
Cipher text generated by second encryption process with XOR different InV using the same plain text as in Figure 18.

**Figure 20:**
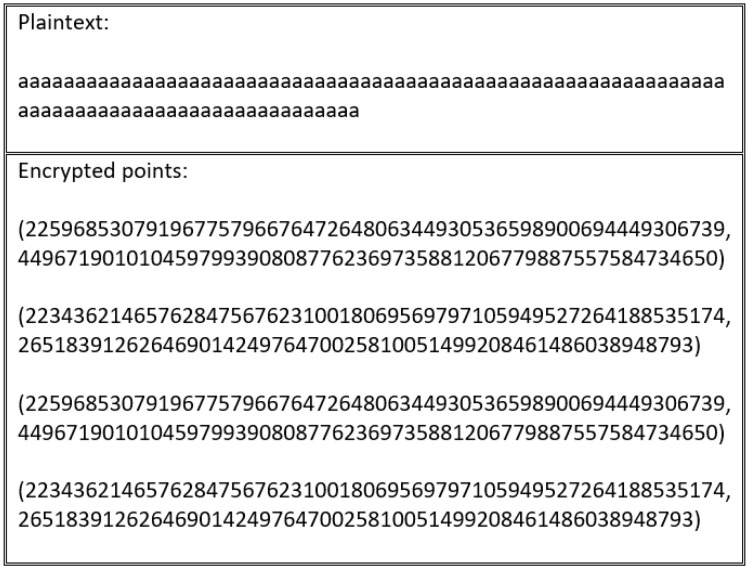
Cipher text generated via encryption of plain text, followed by XORing with InV using a special text that produces the same blocks.

In the scheme proposed by [59], this flaw is resolved by using XOR with InV. Figure 18 shows that the cipher text outputted from the encryption process is different to the cipher text generated by the process depicted in Figure 19, which uses the same plain text. However, these schemes are vulnerable to the same flaw, particularly in the special cases outlined in Figure 20. This flaw occurs when the blocks divided for XOR with InV are equal to $B_{1}=B_{2}=B_{3}=...=B_{n}$. In this case, $B_{1}^{\prime }=InV⊕B_{1}$, $B_{2}^{\prime }=B_{1}^{\prime }⊕B_{2}$, $B_{3}^{\prime }=B_{2}^{\prime }⊕B_{3}$, and so on. Therefore, in the event that all blocks are the same, an attacker can exploit the encryption flaw because XOR with InV operations become:

(6) $$\begin{array}{c} B_{1}^{\prime }=InV⊕B_{1}\\ B_{2}^{\prime }=B_{1}^{\prime }⊕B_{2}\\ B_{2}=B_{1}\to \\ B_{2}^{\prime }=InV⊕B_{1}⊕B_{1}\\ Result\to B_{2}^{\prime }=InV \end{array}$$

This encryption flaw is illustrated in Figure 20.

**Figure 21:**
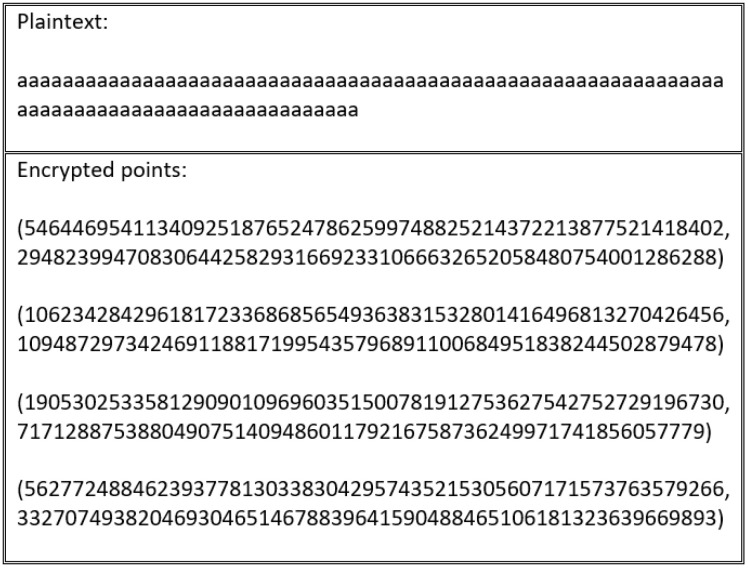
Improved encryption process generating different encrypted points even with the same blocks.

Based on the flaws identified in existing schemes, it is a valuable contribution to the literature to improve the encryption process with CBC. In the proposed scheme, all previous flaws are resolved by using CBC with InV derived from mapping points to secure the cipher text in all forms, as described in Algorithm 5. The encryption operation generated by the optimized encryption process is illustrated in Figure 21.

To evaluate the proposed schemes, security analysis was used with IND-CPA and IND-CCA. In the next subsections, results are given to show that the schemes examined in the literature review are not IND-CPA and IND-CCA. Therefore, it is necessary to prove that the novel scheme proposed in the presented study is both IND-CPA and IND-CCA.

### 4.1. Indistinguishability under Chosen Plain Text Attack IND-CPA

**Figure 22:**
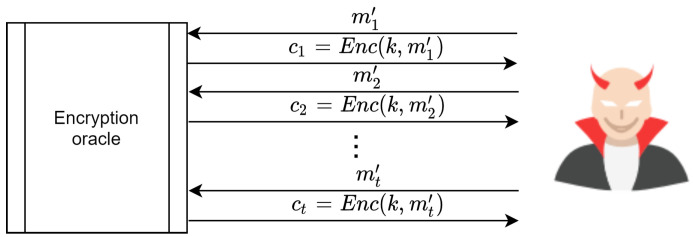
Adversary’s power to submit unlimited messages to encryption oracle in CPA.

A scheme known as IND-CPA if the adversary has the power to submit as many $m_{i,0}$, $m_{i,1}$ i=[1,2,..,q] as desired and also when he or she receives the cipher texts for each message. Then, the adversary must submit two distinct messages, $m_{0}\;and\;m_{1},\;where\;|m_{0}\left|=\right|m_{1}|$, to the encryption oracle, receiving $c\leftarrow Enc(k,m_{b})$. This is illustrated in Figure 22. The challenge is that the adversary is required to guess the value of *b* with a probability of the following:

(7) $$\begin{array}{cc} Adv_{IND-CPA}\left[A,E\right] & =\\ & =|Pr[EXP(0)=1]-Pr[EXP(1)=1]|\\ & =\;negligible \end{array}$$

**Figure 23:**
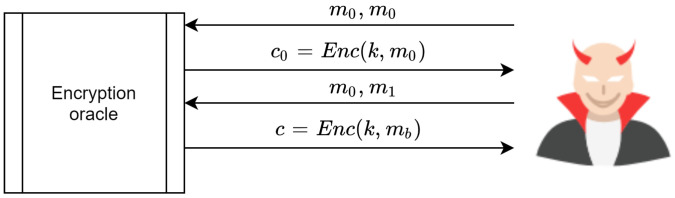
Steps needed for an adversary to win the Chosen Plain Text Attack (CPA) challenge.

Thus, schemes proposed in [55,60,61,62] are insecure under CPA. An adversary can win a challenging game by submitting two identical messages, $m_{0},m_{0}$, to the encryption oracle. In turn, the encryption oracle sends the cipher text $c_{0}$, which is the cipher text for $m_{0}$. In an experiment, the adversary sends two messages, $m_{0},m_{1}$ where $|m_{0}\left|=\right|m_{1}|$, and the encryption oracle responds by encrypting one message and sending one cipher text $c_{b}$. The adversary is then challenged to guess the value of b=0or1, and to determine which cipher text belongs to $m_{0}$ or $m_{1}$. However, the adversary can win the challenge with $Adv_{IND-CPA}\left[A,E\right]=1$. This is because the encryption oracle in such schemes always encrypts the same messages with the same cipher texts and the same key. Therefore, since the adversary already has $c_{0}$, he or she can determine whether the value of *b* is 0 or 1. This is achieved by matching $c_{b}$ with $c_{0}$. If $c_{b}=c_{0}$, then b=0; otherwise, b=1. This process is illustrated in Figure 23.

**Theorem** **1.** *The proposed scheme is (IND-CPA) such that the probability of the adversary to decrypt the message in the two experiments is negligible, as written in Equation (7).*

**Proof.**  This theorem is proven by the fact that the proposed scheme uses a different value of InV for each experiment. Hence, the adversary can submit as many messages as he or she wants to the encryption oracle. The cipher texts generated by the experiment will not be the same, even for the same message. For example, in the second experiment, InV in EXP(0) was different compared to InV in EXP(1). Therefore, when the adversary submits the same message $m_{0},m_{0}$ in EXP(0), he or she will receive the corresponding cipher text in that experiment, which is $c_{0}$. However, when the adversary moves to EXP(1) where the encryption oracle challenges the adversary, the two messages submitted by the adversary $m_{0},m_{1}$ are not distinguishable. This is because the cipher text from $m_{0}$ in EXP(1) is different from the cipher text from the same message $m_{0}$ in EXP(0). For this reason, the probability that the adversary wins the challenge is $Adv_{IND-CPA}\left[A,E\right]=0$

Let Π=(Gen,Enc,Dec) be an ECC encryption scheme, and let us define the experiment between the challenger and the attacker *A* as follows:

- $IND-CPA_{\Pi }\left(A,k\right)$, where *k* is the key size:
  1. The challenger computes $\left(d,PU\right)\leftarrow Gen\left(2^{k}\right)$
  2. The attacker *A* submits 1,2,...,q queries to the challenger to encrypt a plain text of their choice
  3. The attacker *A* sends two messages $\left(m_{0},m_{1}\right)$ for the challenger to encrypt
  4. The challenger computes $\left(c_{b},InV\right)\leftarrow Enc\left(PU,\left(m_{b}⊕InV\right)\right)$
  5. The attacker *A* outputs the value b=0 or b=1, and *A* wins if the probability of *A* guessing a correct value is not negligible

Consider the following games:

**Game (0)**

The attacker *A* makes a request for encrypting a message $\left(m_{0}\right)$, and receives $\left(c_{0},InV\right)\leftarrow Enc\left(PU,\left(m_{0}⊕InV\right)\right)$.

**Game (1)**

The attacker *A* sends two messages $\left(m_{0},m_{1}\right)$, where $m_{0}$ is the same $m_{0}$ as in game 0. The challenger outputs $\left(c_{b},InV^{\prime }\right)\leftarrow Enc\left(PU,\left(m_{b}⊕InV^{\prime }\right)\right)$ to challenge *A* to guess the value of *b*.

**Game (3)**

The attacker *A* returns the value of *b* based on the comparison he or she can perform to distinguish between $\left(m_{0},m_{1}\right)$, which relies on game 0 and 1. However, the probability that *A* achieves this is 0 because the $c_{0}\leftarrow Enc\left(PU,\left(m_{0}⊕InV\right)\right)$ in game 0 is not the same as $c_{0}\leftarrow Enc\left(PU,\left(m_{0}⊕InV^{\prime }\right)\right)$ in game 1. It is also relevant to note that the XOR operation in both games is undertaken with different IV. □

### 4.2. Indistinguishability under Chosen Ciphertext Attack IND-CCA

**Figure 24:**
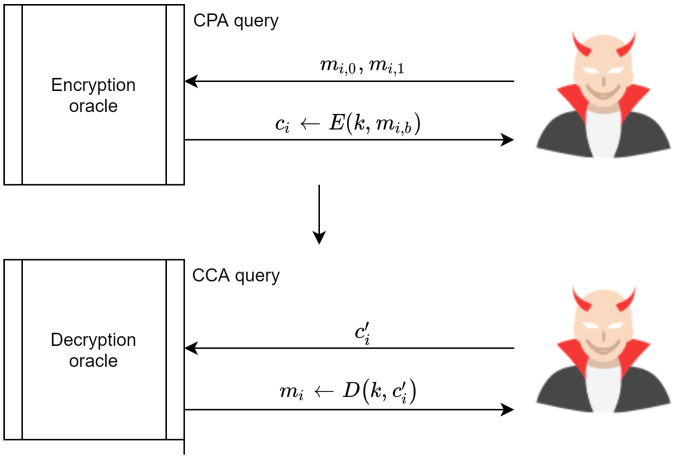
An adversary’s power to submit countless messages to the encryption oracle in CCA.

An encryption scheme is IND-CCA if the adversary has the ability to submit as cipher texts as he wants $c_{i,0}$, $c_{i,1}$i=[1,2,..,q] and to receive the plain text for that cipher text. In addition, the adversary submits any two distinct messages $m_{i,0}$ and $m_{i,1}$, where $|m_{i,0}\left|=\right|m_{i,1}|$ to the encryption oracle and receives $c\leftarrow Enc(k,m_{b})$. This process is depicted in Figure 24. The challenge is that the adversary is required to guess the value of *b* to differentiate between the two experiment’s games, with probability as follows:

(8) $$\begin{array}{cc} Adv_{IND-CCA}\left[A,E\right] & =\\ & =|Pr[EXP(0)=1]-Pr[EXP(1)=1]|\\ & =negligible \end{array}$$

With the above in mind, the scheme proposed in [59] is not secure under CCA. The adversary can win the challenging game by submitting two messages, $m_{0},m_{1}$, to the encryption oracle. In turn, the encryption oracle sends the cipher text $c\leftarrow E\left(k,m_{b}\right)=\left(InV,c_{b}\right)$, which is the cipher text for either $m_{0}$ or $m_{1}$. Subsequently, the adversary modifies *c* by XORing InV with *R*, where R≠0. This produces $c^{\prime }=(InV⊕R,c_{b}$ where $c_{b}=E(k,InV⊕m_{b}$, which is sent to the decryption oracle for decryption.

**Figure 25:**
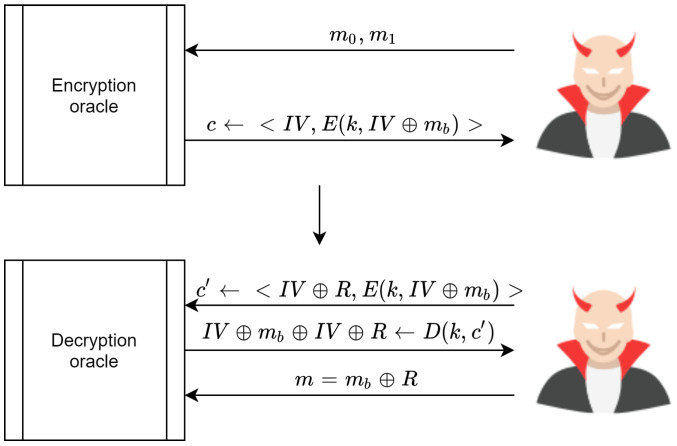
Steps needed for an adversary to win the CCA challenge.

The decryption oracle responds by decrypting the cipher text $D(k,c^{\prime })$. This process relies on the modified InV⊕R. The result is that the decryption process is $m_{b}⊕InV⊕\left(InV⊕R\right)=m_{b}⊕R$. Consequently, an adversary can guess the value of b=0 or b=1 by XORing $m_{b}⊕R$, after which it can be compared directly with the received message $m_{b}$. Therefore, the adversary can win the challenge with $Adv_{IND-CCA}\left[A,E\right]=1$. This process is illustrated in Figure 25.

**Theorem** **2.** *The proposed scheme is indistinguishable under IND-CCA such that the probability of the adversary decrypting the message in the two experiments is negligible, as written in Equation (8).*

**Figure 26:**
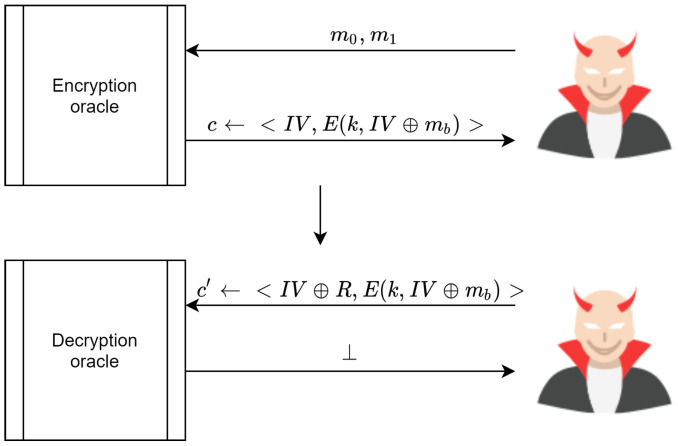
Proposed scheme IND-CCA proof.

**Proof.**  The proof for $Adv_{CCA}\left[B_{1},E\right]$ starts by noting the adversary’s ability to access both the encryption and decryption oracle. At the same time, the adversary cannot submit $c_{i}$ received from the encryption oracle and then submit it for decryption to distinguish the experiment game. However, the adversary can modify the received $c_{i}\leftarrow E\left(k,m_{i}\right)$ and submit it for decryption as the modified $c_{i}^{\prime }\neq c_{i}$. Thus, the adversary can win the challenging game by XORing InV with a random R, thereby guessing $m_{i}$ (see Figure 25). The proposed scheme offers integrity to cipher texts. It also protects transmitted cipher texts from tampering. Therefore, the decryption oracle drops any modified $c_{i}$, meaning that the oracle responds to the adversary with $⊥\leftarrow <InV⊕R,c_{i})>$ for every modified message, as illustrated in Figure 26. Resultantly, the proposed scheme is negligible under Equation (8), meaning that it is indistinguishable under IND-CCA.

Let Π=(Gen,Enc,Dec,Sign,Ver) be an ECC encryption scheme, and let us define an experiment between the challenger and attacker *A* as follows:

- $IND-CCA_{\Pi }\left(A,k\right)$, where *k* is the key size:
  1. The challenger computes $\left(d,PU\right)\leftarrow Gen\left(2^{k}\right)$
  2. The attacker *A* submits 1,2,...,q queries to the challenger to decrypt their chosen cipher text
  3. The attacker *A* sends two messages $\left(m_{0},m_{1}\right)$ to the challenger for encryption
  4. The challenger computes $\left(c_{b},InV,Sign\right)\leftarrow Enc\left(PU,\left(m_{b}⊕InV\right)\right)$
  5. The attacker *A* outputs the value of b=0 or b=1, where *A* wins if the probability of guessing *A* correctly is not negligible

Consider the following games:

**Game (0)**

The attacker *A* makes a request for encrypting a message $\left(m_{0}\right)$, and receives $\left(c_{0},InV,Sign\right)\leftarrow Enc\left(PU,\left(m_{0}⊕InV\right)\right)$.

**Game (1)**

The attacker *A* modifies the cipher text from game 0 $\left(c_{0},InV,Sign\right)$ to XOR the InV with a random value R≠0. A request is made for the challenger to decrypt the cipher text $\left(c_{0},InV⊕R,Sign\right)$.

**Game (3)**

The attacker *A* can compute the plain text from $\left(m_{b}⊕InV⊕\left(InV⊕R\right)\right)\leftarrow Dec\left(c_{0},InV⊕R,Sign\right)$, which results in $\left(m_{b}⊕R\right)$. In this case, *A* can compare $\left(m_{b}⊕R\right)$ with $\left(m_{0}⊕R\right)$ and $\left(m_{1}⊕R\right)$. However, *A* will fail to win because the challenger will discard the modified $\left(c_{0},InV⊕R,Sign\right)$. This stems from the fact that Sign is invalid, meaning that probability of the success of *A* is 0. □

### 4.3. Malleability Attack

**Figure 27:**
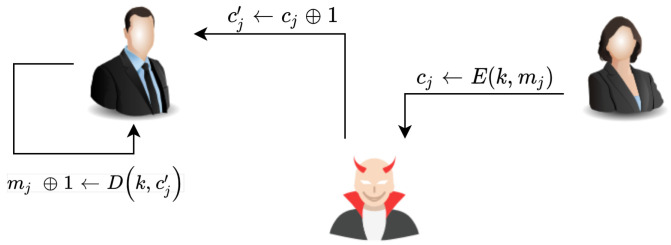
Overview of the malleability attack.

Changing encrypted data can lead to the modification of the plain text after decryption, which is known as a malleability attack. For instance, the attacker modifies the InV with the value of 1, which means that the first block of the plain text message will be XORed with 1. As a result, the reset of blocks in the CBC will also be modified. The steps involved in the malleability attack are depicted in Figure 27.

**Theorem** **3.** *The proposed scheme is resistant to the malleability attack.*

**Figure 28:**
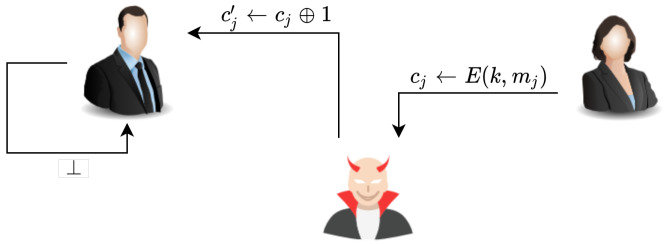
Resistance of the proposed scheme to the malleability attack.

**Proof.**  It is known that an adversary can eavesdrop on the messages sent between the two parties. Adversaries can also change messages and return them to either party. However, the recipient checks the received message’s integrity before decrypting the cipher text. Therefore, the recipient ignores the received message because it has an invalid signature. An illustration of this proof is given in Figure 28

□

A security analysis comparison was undertaken between the scheme proposed in this study and others mentioned in the literature (see Table 2).

### 4.4. Performance Evaluation

The main goal of the proposed scheme was to provide a secure scheme that resolved encryption flaws that yield to CPA and CCA attacks. The study also sought to offer a scheme with a suitable level of performance in constrained environments. The security aspect of the proposed scheme was proven in the previous section, and this section evaluated the performance of the scheme based on two criteria: enhancing the process of mapping points to an elliptic curve; and time, space overhead, and power consumption in a simulation environment.

#### 4.4.1. Mapping Points to an Elliptic Curve

**Figure 29:**
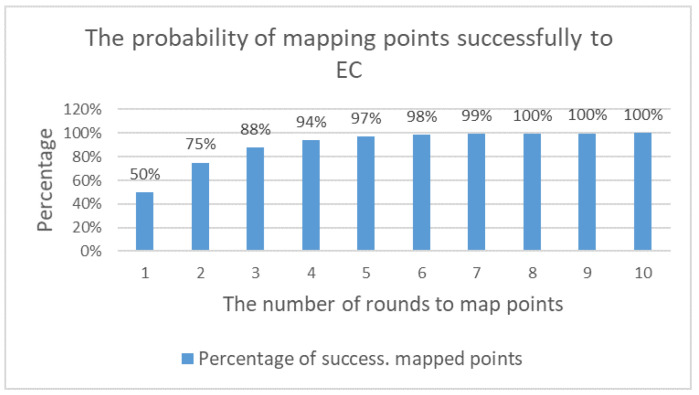
Probability of successfully mapping points to an elliptic curve based on number of rounds.

Mapping points to an elliptic curve should be undertaken correctly and efficiently. As illustrated in Figure 29, 50% of $x_{i}$ points cannot be mapped to the EC as there is no $y_{i}$ axis that meets the EC’s equation. Therefore, it is necessary to increase the value of $x_{i}$ by 1, and then to recompute $y_{i}$ until a value is found that matches. As a result, the $x_{i}$ point is eventually mapped. In many schemes, the characters in plain text are converted into numerical values based on the ASCII table, which facilitates their mapping onto the elliptic curve. Therefore, when these values are not mapped from the beginning, the value to be mapped increases. However, this step changes the original value, and the plain text is lost. For this reason, to overcome this problem, two methods are widely used to map points: the probability method and the appending method.

In the probability method, which was introduced by King (2009) [64], the value of *k* is defined, which represents the number of rounds needed to map the points. This value is multiplied by $x_{i}$, and the product is used in the mapping phase with ability to increment it in *k* rounds. In turn, the value of $x_{i}$ can be restored by calculating the value of the mapped point: $\left\lfloor \frac{mapped\;point}{k}\right\rfloor$. In the second method, the appending method, $x_{i}$ is appended by number of bits representing the number of rounds required. For instance, if the scheme defines the appended bits as 000, then the number of rounds that can be safely used in the mapping phase is $2^{3}=8$ rounds.

**Figure 30:**
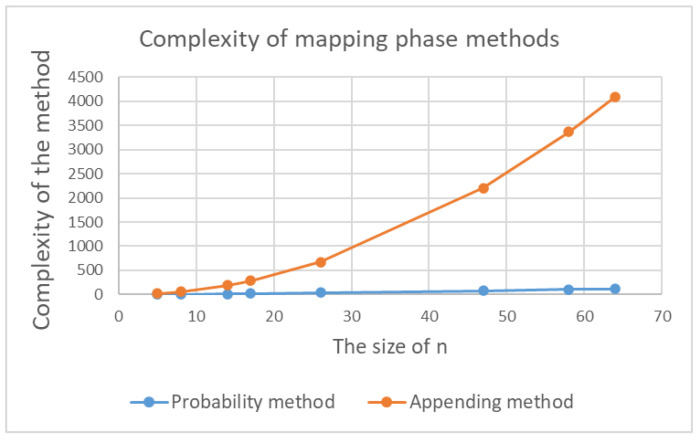
Complexity comparison of probability and appending mapping methods.

Each method has advantages and disadvantages relating to computational efficiency and the maximum number of rounds. The probability method is more efficient and less complex than the appending method. Using the Harvey-Hoeven algorithm [65], the multiplication operation complexity is O(nlogn), where *n* is the numerical value size in bits. However, the complexity of appending two texts (i.e., concatenations) is denoted as $O(n^{2})$ [66], which highlights the fact that the appending method is more complex than the probability method. This is illustrated in Figure 30.

**Figure 31:**
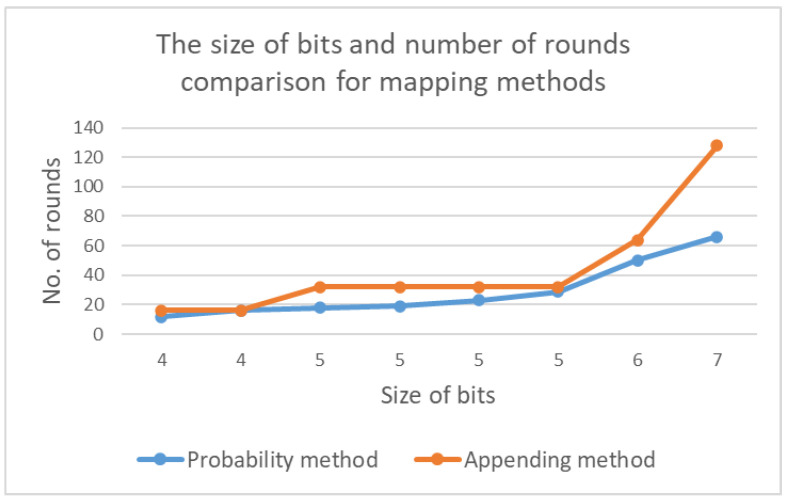
Comparison of number of rounds used in mapping phase method and the size of bits added to $x_{i}$.

The appending method guarantees to provide a maximum number of rounds base on the appended bits. As the appending methods concatenate fixed bits *b* to the corresponding binary $x_{i}$ value, $x_{i}$ can be incremented $2^{b}$ times. In contrast, the probability method increases the same size of bits, but in many cases, it would allow fewer round increments compared to the appending method. This is outlined in Figure 31.

**Figure 32:**
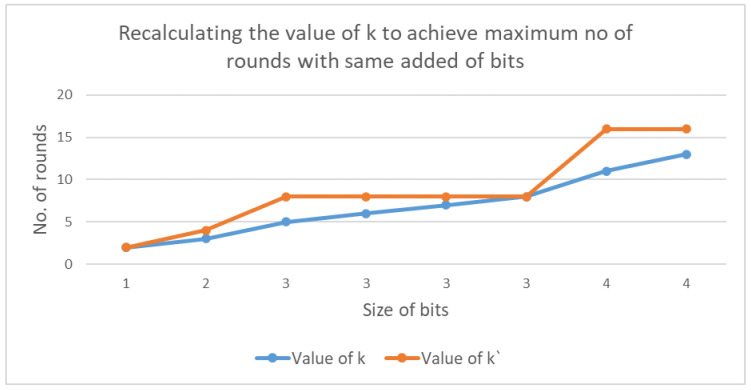
Recalculating $k^{\prime }$ to increase the maximum number of rounds to the size of added bits.

In the proposed scheme, the probability method was used to improve performance. Hoevewr, to increase the efficiency of the choice of *k*, the proposed scheme took advantage of the appending method to select this value. This was achieved by recalculating $k^{\prime }=2^{logk}$, which was used to set the number of rounds to the maximum value of the extra bits added to the value of $x_{i}$, as shown in Figure 32.

**Figure 33:**
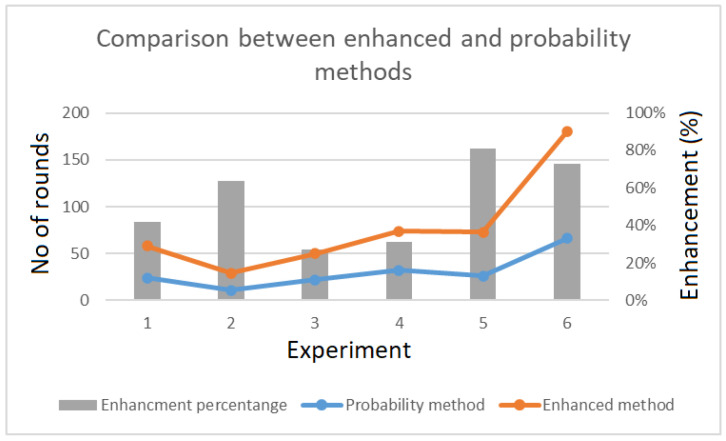
Percentage improvement between the enhanced selection of *k* provides in comparison with probability method.

The enhancement made to the probability method increases the number of mapping rounds with same size of the padding bit. As a result, the efficiency was more than 80% in some cases. Figure 33 shows the percentage of improvement that the proposed enhanced selection of *k* method offers compared to the probability method.

#### 4.4.2. Simulation Performance Evaluation

The performance evaluation of the proposed scheme focused on whether the enhancement of the encoding and mapping phases, which was intended to improve resistance to encryption attacks, negatively affected performance. Therefore, the performance of the proposed scheme against other schemes took place to evaluate time overhead, space usage, and power consumption. The schemes are divided to three groups: schemes that did not use InV or any other methods to manipulate the mapped points as [55,60,61,62]; schemes that used fixed InV to overcome CPA, including [59]; and finally, schemes that used CBC to overcome the CCA (in this case, only the proposed scheme).

**Figure 34:**
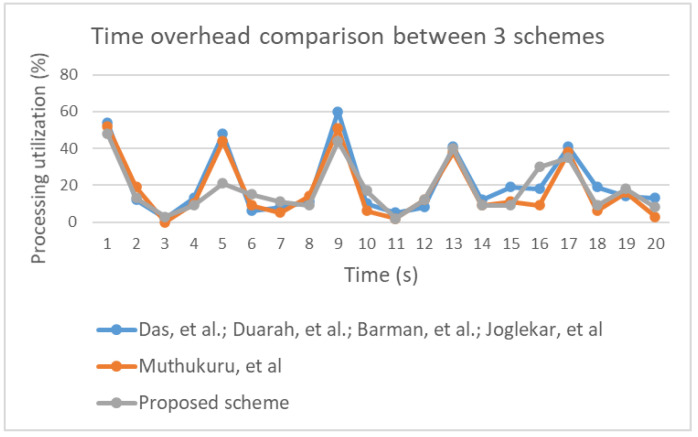
Comparison of time overhead between the proposed scheme and other schemes.

**Figure 35:**
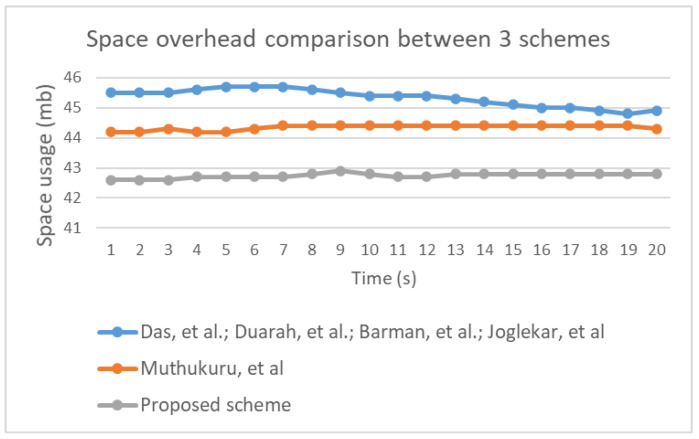
Comparison of space usage between the proposed scheme and other schemes.

**Figure 36:**
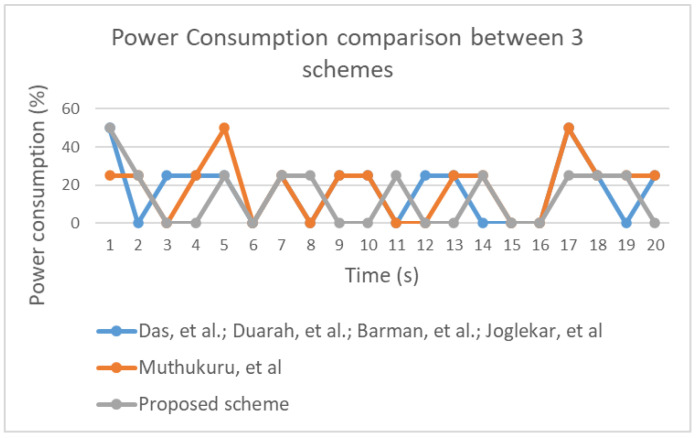
Comparison of power consumption between the proposed scheme and other schemes.

The performance evaluation involved running an experimental simulation 5 times for 20 s each using a low computation device. Figure 34 illustrates time overhead in the three groups included in the performance evaluation. Notably, all three groups were associated with similar levels of utilization. Therefore, it is reasonable to conclude that improving the security of the proposed scheme did not affect the time overhead. Space usage variation was identified during the experiment, as illustrated in Figure 35. Additionally, power consumption for each group was determined based on values (high, medium, low, or none), which are shown in Figure 36. The results indicate that power consumption was similar across the three groups.

It is worth mentioning that, the proposed scheme was simulated, tested and compared to the state of the art schemes in a simulated environment using an Android Virtual Device (AVD). AVD has variety of low computation devices images built by Android OS, and it provides processing space and power consumption monitoring and logging. In addition, we used the Bluej Java Development Environment to code the proposed scheme. The platform used to host the AVD is based on Windows 10, and, in terms of hardware, an Intel Core i7-4510U was used with a 128 GB SSD and 8 GB RAM.

## 5. Conclusions and Future Research

This study proposes a novel approach to elliptic curve cryptography (ECC) that offers AE properties to secure cipher text and to enhance the encoding of text effectively and map the encoded text to an elliptic curve. Previous schemes neglect to consider the importance of the encoding phase, which makes them vulnerable to attack. Therefore, this study focused on the encoding phase, seeking to secure it against several encryption attacks, including CPA, CCA, and malleability attacks. This study also undertook a security analysis to present a proof for the resistance of the proposed scheme against specific encryption attacks. Additionally, the study conducted a performance evaluation to compare the impact of the security enhancement of the proposed scheme on time overhead, space usage, and power consumption to other schemes. The simulation experiment shows that the proposed scheme performed just as well as the other schemes, with no noticeable increase in computation overhead. As a result, the proposed scheme outperforms the security of other schemes and maintains the same computational overhead.

In future research, the authors intend to implement the proposed scheme in different environments such as enhance the protection using TPM-based for mobile agents. Moreover, adapt the scheme in Monitoring the cloud computing architecture to enhance Dynamic Security Properties. In addition, apply the proposed scheme with Policy Based Management to increase the Security of Cloud Computing. These application of proposed scheme lead to study the security analysis and performance evaluation in comparison with other similar schemes. Additionally, more security properties may be added to the study to increase the security requirements, which might be required in new environments.

## Figures and Tables

**Table 1:** Notation relevant to the proposed scheme.

Notation	Description
$id_{edge}$	Edge identification number
$id_{ni}$	Node identification number
$d_{edge}$	Edge private key
$d_{ni}$	Node private key
*G*	EC base point
$PU_{edge}$	Edge public key = $d_{b}*G$
$PU_{ni}$	Node public key = $d_{ni}*G$
*p*	Large prime number (192-bit)
a,b	EC coefficients, s.t. $4a^{3}+27b^{2}\;mod\;p\neq 0$
$y^{2}\equiv x^{3}+ax+b\;mod\;p$	EC map points equation
$H_{1}$	Hash function used only by edge
nList	Node list containing references and $H_{1}\left(id_{ni}\right)$
HASH	Signing message $C_{M}$ hash function
$k_{sh}$	Shared group key
$gk_{sh}$	Shared group point ($k_{sh}\times G$)
InV	Random initial vector (192-bit)
PRK	Private random key (192-bit)
*k*	Random integer chosen from [1,p−1]
$C_{M}$	Cipher text (all encrypted points)
⊕	XOR used in mapping phase to secure mapped points
+	Addition operation used in ECC to encrypt mapped points with $gk_{sh}$

**Table 2:** Security analysis comparison of proposed scheme and other schemes.

	1	2	3	4	5	6	7	8
Barman, [60]	N	N	N	N	N	O(nlogn)	*n*	N
Joglekar, [62]	N	N	N	N	N	N/A	N/A	N
Muthukuru, [59]	Y	N	N	N	N	O(nlogn)	*n*	N
Duarah, [61]	N	N	N	N	N	O(nlogn)	*n*	N
Das, [55]	N	N	N	N	N	N/A	N/A	N
Proposed scheme	Y	Y	Y	Y	Y	O(nlogn)	$2^{n}$	Y

1. IND-CPA; 2. IND-CCA; 3. Resistance to malleability attack; 4. Integrity; 5. Authenticated encryption; 6. Complexity of mapping phase; 7. No of rounds based on *n* appending bits; 8. Offers nonrepudiation

## Abbreviations

The following abbreviations are used in this manuscript:

ECC	Elliptic Curve Cryptography
IoT	Internet of Things
InV	Initial Vector
CBC	Cipher Block Chaining
AE	Authenticated Encryption
CPA	Chosen Plaintext Attack
CCA	Chosen Ciphertext Attack
ECIES	Elliptic Curve Integrated Encryption Scheme
ECDLP	Elliptic Curve Discrete Logarithm Problem
